# Distinct trajectories of PDI-measured dignity-related distress and associated factors Among ICU survivors during early post-ICU recovery: a prospective cohort study

**DOI:** 10.3389/fmed.2026.1825732

**Published:** 2026-10-29

**Authors:** Hongping Tang, Yulian Wu, Jin Yang, Min Wang, Ling Lei, Chencong Nie, Xiaoling Wu

**Affiliations:** Zigong Fourth People’s Hospital, Zigong, China

**Keywords:** dignity-related distress, ICU survivors, latent class growth analysis, post-ICU recovery, symptom trajectory

## Abstract

**Background:**

With the increasing survival of critically ill patients, attention has shifted toward the long-term psychological outcomes of ICU survivors. Dignity-related distress may represent an important but under-recognized aspect of psychological recovery after critical illness. However, longitudinal patterns of PDI-measured dignity-related distress during early recovery after ICU transfer remain poorly understood.

**Objective:**

This study aimed to identify distinct trajectories of PDI-measured dignity-related distress among ICU survivors and to explore factors associated with different trajectory patterns.

**Methods:**

A prospective cohort study was conducted among ICU survivors from a tertiary hospital in Sichuan Province, China. The Patient Dignity Inventory (PDI) was administered at four time points after ICU transfer: at transfer (T1), 3 days (T2), 7 days (T3), and 14 days (T4). Latent class growth analysis (LCGA) was used to identify heterogeneous trajectories of PDI-measured dignity-related distress. Least absolute shrinkage and selection operator (LASSO) regression was applied for variable selection, followed by multivariable logistic regression to examine factors associated with trajectory class membership after multivariable adjustment.

**Results:**

A total of 305 ICU survivors meeting the eligibility criteria were initially enrolled, and 284 patients who completed all follow-up assessments were included in the final analysis. Two distinct trajectories of PDI-measured dignity-related distress were identified: a low-level gradual improvement trajectory (73.94%) and a high-level rapid improvement trajectory (26.06%). Although PDI-measured dignity-related distress decreased over time in both groups, patients in the high-level trajectory presented with significantly higher initial symptom levels. Multivariate logistic regression analysis showed that age (OR=1.103, 95% CI: 1.038–1.173), Clinical Frailty Scale (CFS) score (OR=0.560, 95% CI: 0.336–0.932), ICU length of stay (OR=1.199, 95% CI: 1.028–1.398), number of combined systemic dysfunctions (OR=2.180, 95% CI: 1.123–4.232), and primary diagnosis were significantly associated with trajectory class membership.

**Conclusion:**

Among communicative and cognitively intact ICU survivors included in this cohort, exploratory analysis identified heterogeneous trajectories of PDI-measured dignity-related distress during early recovery after ICU transfer. These findings may help characterize variation in dignity-related psychological recovery and generate hypotheses for future studies of supportive care in this population.

## 1 Introduction

The intensive care unit (ICU) is the core setting for the treatment of critically ill patients. Although intensive monitoring and advanced medical interventions in the ICU can effectively safeguard patients’ lives, they may also exert profound effects on patients’ physical and psychological well-being (1). During ICU treatment, patients often undergo invasive procedures such as mechanical ventilation, tracheal intubation, and physical restraint. While these interventions are clinically necessary, they may also lead to loss of autonomy, exposure of personal privacy, and alterations in self-image, which can subsequently affect patients’ sense of dignity (2). These physiological and psychological challenges may place ICU patients at increased risk of experiencing dignity-related distress. Dignity, as a core psychological state reflecting an individual's sense of self-respect, personal value, and being respected by others, plays an important role in patients’ recovery and overall quality of life (3). Dignity-related distress may involve diminished self-worth, reduced autonomy, impaired self-identity, or difficulties in maintaining personal value due to physical, psychological, or social challenges during illness (4). Therefore, preserving dignity represents an important component of recovery among ICU survivors, and understanding changes in dignity-related experiences during the post-ICU period may provide valuable insights into patients’ psychological adaptation and supportive care needs.

Importantly, dignity-related distress should not be considered synonymous with other commonly assessed outcomes after critical illness. Anxiety and depressive symptoms primarily reflect emotional distress, functional dependence describes limitations in patients’ ability to perform daily activities, and post-intensive care syndrome encompasses a broader constellation of physical, cognitive, and psychological sequelae after critical illness (5). In contrast, dignity-related distress concerns patients’ perceived self-worth, autonomy, personal identity, sense of control, and ability to maintain personal value in the context of illness and care (6). Although these constructs may coexist and interact during post-ICU recovery, dignity-related distress may capture a patient-centered dimension of recovery that is not fully represented by conventional psychological, functional, or quality-of-life outcomes. Assessing dignity-related distress may therefore provide complementary information regarding patients’ psychological adaptation and dignity-conserving care needs during the transition from the ICU to the general ward. In the present study, these dignity-related experiences were operationalized using the Patient Dignity Inventory (PDI), which assesses dignity-related distress rather than the broader construct of dignity itself. Accordingly, the term “PDI-measured dignity-related distress” is used when referring specifically to the study outcome.

Longitudinal evidence regarding dignity-related distress during early post-ICU recovery remains limited. Existing studies have more commonly described the overall level of dignity-related distress or examined factors associated with dignity-related distress at a single assessment point (7). Such approaches provide important information on symptom burden but may not adequately characterize heterogeneity in recovery over time. In particular, changes in group-level mean scores can describe the average direction of symptom improvement or deterioration while potentially obscuring subgroups of patients who begin with different levels of dignity-related distress or recover at different rates. A trajectory-based approach can therefore provide additional information by identifying groups of patients with distinct longitudinal patterns rather than assuming that all ICU survivors follow a similar recovery course. Characterizing such heterogeneity may help identify patients with persistently high or rapidly changing dignity-related distress and provide a more clinically informative basis for individualized supportive care.

Accordingly, the present study focused on longitudinal changes in PDI-measured dignity-related distress symptoms among ICU survivors during the early post-ICU recovery period. The primary objective was to identify distinct trajectories of PDI-measured dignity-related distress across multiple assessment points using latent class growth analysis (LCGA). The secondary objective was to examine demographic and clinical factors associated with trajectory class membership. Characterizing heterogeneous patterns of PDI-measured dignity-related distress over time may provide insight into differences in early psychological recovery after ICU transfer.

## 2 Materials and methods

### 2.1 Participants

This prospective cohort study included patients transferred from the medical ICU, surgical ICU, emergency ICU, and other adult ICUs of a tertiary Grade A general hospital in Sichuan Province between October 2025 and February 2026. The study aimed to investigate early trajectories of PDI-measured dignity-related distress among ICU survivors. All participants were transferred from the ICU in accordance with internationally recognized ICU discharge criteria (8), including stable vital signs, liberation from mechanical ventilation, clear consciousness without delirium, stable organ function, and no requirement for ongoing ICU-level monitoring or support.

The inclusion criteria were as follows: (1) adult ICU survivors aged ≥18 years who were alive at ICU transfer and transferred to general wards, with clear consciousness and the ability to communicate effectively either verbally or non-verbally, as confirmed by an Intensive Care Delirium Screening Checklist (ICDSC) score of 0 at T1 (the time of transfer from the ICU to the general ward); (2) an ICU stay of ≥24 h; and (3) voluntary participation with written informed consent. The exclusion criteria were as follows: (1) a history of severe psychiatric disorders (e.g., depression or delirium) within the previous three months; and (2) participation in other studies related to mental health or emotional status, or the presence of severe cognitive impairment, visual or hearing impairment, or other conditions that prevented completion of the questionnaire.

During the recruitment period, all patients who met the predefined inclusion and exclusion criteria were consecutively enrolled, resulting in a total of 305 eligible participants at T1. Because the total number of ICU patients assessed before application of the eligibility criteria was not prospectively recorded, the overall number screened and the number excluded before enrolment could not be reliably reconstructed. Participant enrolment, follow-up, and inclusion in the final analysis are summarized in Figure 1.

**Figure 1:**
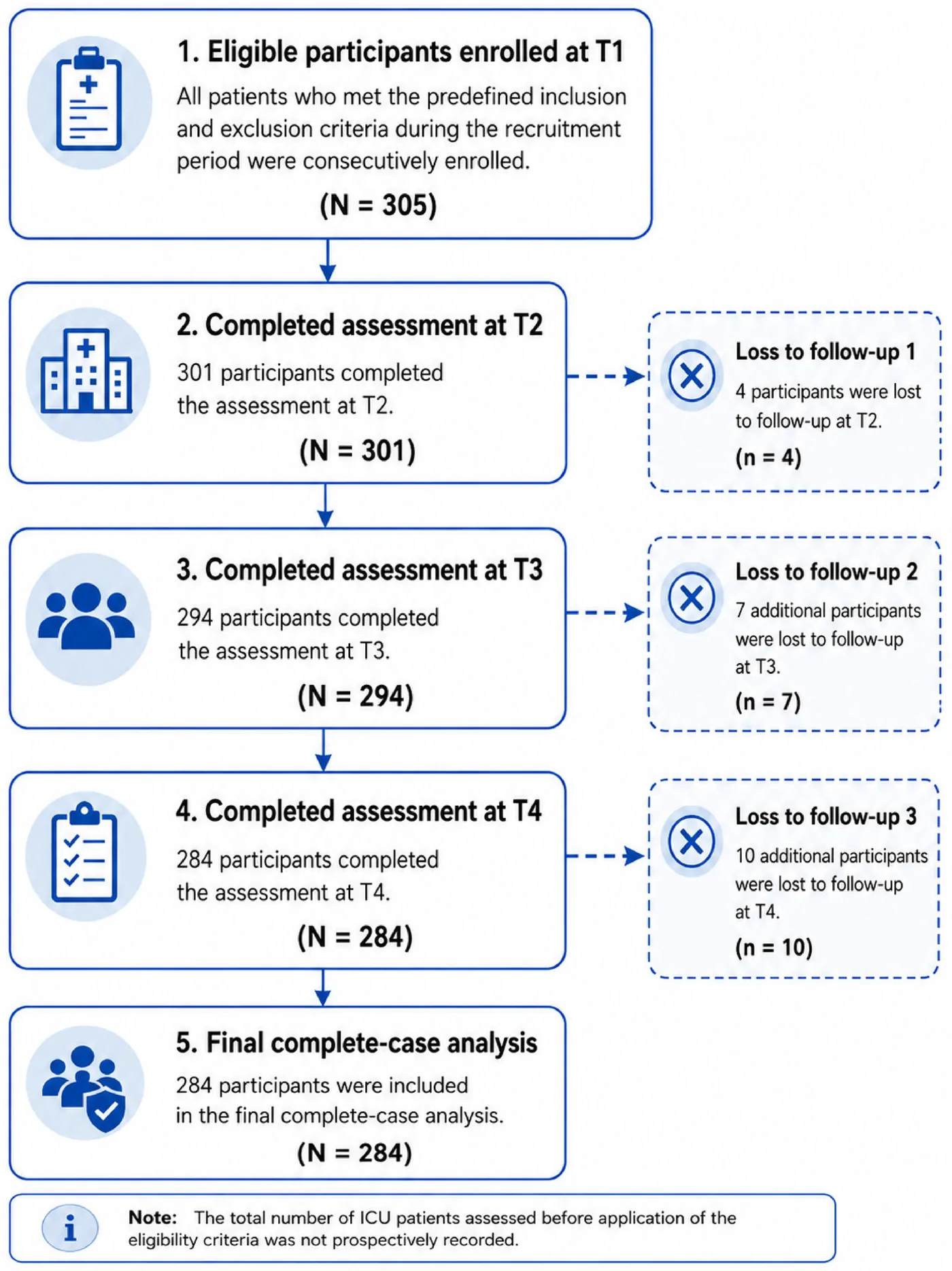
Flow diagram of participant enrolment, follow-up, and final analysis.

### 2.2 Sample size

The sample size was estimated using the formula $N={\left(\frac{Z\alpha /2\cdot \sigma }{E}\right)}^{2}$, where Z_a/2_ was set at 1.96 (corresponding to a significance level of 0.05), σ represented the population standard deviation, and E was the width of the confidence interval (9). Based on preliminary survey results from patients meeting the inclusion and exclusion criteria, the estimated σ was approximately 22. To ensure high precision in the research findings, the confidence interval width was set at ±3 units. Substituting these values into the formula, it was determined that approximately 206 patients would need to be surveyed. Considering a 10% rate of invalid samples, the final sample size required was calculated to be 227 patients.

Considering the planned longitudinal trajectory analysis, adequate sample size is also important for reliable latent class estimation. Previous simulation studies have demonstrated that sample size requirements for latent class growth models depend on multiple factors, including the number of latent classes, measurement occasions, class proportions, and the degree of class separation (10). In this study, 284 participants who completed four repeated assessments were included in the final trajectory analysis. The final two-class model demonstrated reasonable class proportions and high classification accuracy (entropy = 0.994), providing supportive evidence for the classification quality and interpretability of the identified trajectory solution.

### 2.3 Ethics approval and informed consent

This prospective study was conducted under a research protocol approved by the Ethics Committee of Zigong Fourth People's Hospital, Sichuan Province, on March 7, 2024 (Approval No. EC-2024-008), with approval valid until March 7, 2026. The approved protocol specified the prospective study design, target population, use of the Patient Dignity Inventory, and a longitudinal methodological framework developed with reference to previous literature. The specific assessment occasions used in the present study—at ICU transfer (T1), 3 days after transfer (T2), 7 days after transfer (T3), and 14 days after transfer (T4)—were operationalized within this approved methodological framework. Participant recruitment began in October 2025 and was completed in February 2026, within the approved study period.

All participants provided written informed consent before participation after receiving a full explanation of the study objectives, procedures, potential risks, and benefits. Given that all enrolled participants had sufficient cognitive and communication capacity at the time of ICU transfer, informed consent was obtained directly from the patients themselves. Participation was entirely voluntary, and participants were informed that they could withdraw from the study at any time without any adverse effect on their medical treatment or care. All collected data were kept confidential and used solely for academic research and statistical analysis.

### 2.4 General information questionnaire

A general information questionnaire was designed by the research team based on a review of relevant literature and clinical practice. The questionnaire was used to collect patients’ demographic characteristics and disease-related information. Demographic variables included age, gender, education level, marital status, and employment status. Clinical and treatment-related variables included type of ICU (medical ICU, surgical ICU, emergency ICU, and other ICUs), ICU length of stay, frequency of ICU admissions, defined as the cumulative number of times that the patient had been admitted to an ICU since birth, primary diagnosis categorized according to the primary affected organ system (including nervous system, respiratory system, circulatory system, digestive system, and other systemic diseases), Acute Physiology and Chronic Health Evaluation II (APACHE II) score recorded within 24 h of ICU admission, Clinical Frailty Scale (CFS) score assessed at ICU admission, number of combined systemic dysfunctions, and relevant treatment measures (e.g., mechanical ventilation therapy and enteral nutrition therapy). The CFS was used to assess patients’ frailty status based on their functional level and clinical condition before ICU admission. It is a 9-point scale ranging from 1 (very fit) to 9 (terminally ill), with higher scores indicating a greater degree of frailty (11).

### 2.5 Patient dignity inventory

The Patient Dignity Inventory (PDI), originally developed by Chochinov et al., was used to assess dignity-related distress in the present study (5). The original instrument contains 25 items covering multiple sources of dignity-related distress, including symptom distress, existential distress, dependency, peace of mind, and social support. Permission to use the original English-language PDI was obtained from the original developer. Permission was also obtained from the authors of an existing Chinese adaptation. For the present study, a study-specific Chinese-language adaptation was prepared from the original English PDI, with reference to the existing Chinese version to improve linguistic clarity and cultural appropriateness for mainland Chinese participants. Linguistic and cultural adjustments were limited to wording intended to enhance comprehensibility and cultural relevance and did not intentionally alter the core meaning or essential content of the original 25 items or the scoring framework.

Previous studies have applied the PDI in critically ill and ICU populations, providing an empirical basis for its exploratory use in critical-care settings (12). However, prior use in critically ill populations does not establish full construct validity or measurement equivalence in post-ICU survivors. In the present study, participants were restricted to individuals who were alert, communicative, and cognitively capable of completing the questionnaire after ICU transfer. Each item was rated on a 5-point Likert scale from 1 to 5, yielding a total score ranging from 25 to 125, with higher scores indicating greater PDI-measured dignity-related distress. The total score was used for longitudinal trajectory analysis. The PDI demonstrated good internal consistency in the present sample (Cronbach's *α* = 0.924); however, formal construct validation of the study-specific Chinese-language adaptation in post-ICU survivors was not performed.

### 2.6 Data collection

Patients were followed up and assessed at four predefined time points after ICU transfer. Specifically, data were collected at four time points: at ICU transfer (T1), 3 days after transfer (T2), 7 days after transfer (T3), and 14 days after transfer (T4). At each assessment point, dignity-related distress was assessed using the Patient Dignity Inventory (PDI).

All data collection procedures were conducted by trained members of the research team. Prior to participation, all patients provided written informed consent after receiving a full explanation of the study purpose and procedures. Data were collected using face-to-face assessments during hospitalization. For patients who had been discharged from the hospital, follow-up assessments were conducted through telephone interviews, home visits, or outpatient visits according to patients’ availability and follow-up arrangements. During each assessment, patients completed the Patient Dignity Inventory (PDI) based on their personal experiences, and research staff provided assistance when necessary to ensure accurate completion of the questionnaires.

### 2.7 Quality control

To ensure the accuracy and reliability of the data, strict quality control measures were implemented throughout the data collection process. First, all researchers received standardized training prior to data collection. The training covered the study background, data collection procedures, and questionnaire administration methods. After the training, the researchers’ understanding was evaluated through an assessment to ensure that each investigator involved in data collection possessed sufficient knowledge and skills.

During the data collection process, questionnaires for each participant were completed and checked independently by two researchers to ensure accurate data entry. To minimize errors caused by misunderstanding or language differences, on-site guidance was provided to patients when necessary to ensure that the questionnaires were completed correctly. For questionnaires that were incomplete or did not meet the requirements, the research team verified and corrected the information with the patients in a timely manner.

To further ensure data quality, all questionnaires were cross-checked by two researchers after each round of data collection. Participants who did not complete all four scheduled assessments were not included in the primary complete-case longitudinal analysis. In addition, random audits of the collected data were conducted by the research team to ensure the standardization and consistency of the data collection process. All data were compiled and analyzed collectively after completion of data collection to ensure the reliability and scientific rigor of the study results.

### 2.8 Statistical analysis

Data analysis was conducted using SPSS 26.0, Mplus 8.7, and R software, including latent class growth analysis, variable selection, and multivariate regression analysis, with a two-tailed significance level set at *p* < 0.05 for all statistical tests. To assess potential attrition bias, baseline characteristics, including age, sex, and primary diagnosis, as well as the T1 PDI total score, were compared between participants who completed all four assessments and those who did not complete the follow-up. Continuous variables were compared using the independent-samples t test or Mann–Whitney U test, as appropriate, whereas categorical variables were compared using the chi-square test or Fisher's exact test. The primary longitudinal analysis was conducted using complete cases. Of the 305 participants enrolled at T1, 21 (6.9%) did not complete all four assessments. Because the proportion of incomplete follow-up was relatively low and no statistically significant baseline differences were identified between completers and non-completers for age, sex, primary diagnosis, or T1 PDI score, no additional imputation-based sensitivity analysis was performed.

For trajectory classification, latent class growth analysis (LCGA) was performed using Mplus to identify heterogeneous longitudinal patterns of PDI-measured dignity-related distress across the four assessment occasions. The assessment times were coded according to the actual number of days after ICU transfer as 0, 3, 7, and 14 for T1, T2, T3, and T4, respectively. Both linear and quadratic growth functions were examined. In accordance with the LCGA framework, the within-class variances of the intercept, linear slope, and quadratic growth factors were fixed at zero. Residual variances at each measurement occasion were freely estimated and constrained to be equal across latent classes. Because the repeated dignity scores showed evidence of non-normality, the robust maximum likelihood estimator (MLR) was used. To reduce the risk of convergence to local maxima, 1,000 random sets of starting values were used, with 250 retained for final-stage optimization. Model convergence was evaluated using the default Mplus convergence criteria and was further confirmed by normal model termination and replication of the best log-likelihood value.

Model fit was evaluated using the Akaike Information Criterion (AIC), Bayesian Information Criterion (BIC), sample-size adjusted Bayesian Information Criterion (aBIC), entropy, the Lo–Mendell–Rubin likelihood ratio test (LMR), and the bootstrap likelihood ratio test (BLRT). Lower AIC, BIC, and aBIC values indicated better model fit, whereas higher entropy values indicated greater classification accuracy. Average posterior class probabilities were additionally examined to assess classification quality. Model selection was based on statistical fit indices, classification accuracy, class proportions, model stability, parsimony, and clinical interpretability. Comparison of the linear and quadratic two-class specifications indicated better fit for the quadratic model; therefore, the quadratic LCGA specification was retained for the final trajectory analysis.

After identification of the trajectory classes, least absolute shrinkage and selection operator (LASSO) regression was performed using the glmnet package in R to screen candidate variables associated with trajectory class membership. All 30 prespecified demographic, socioeconomic, clinical, and treatment-related variables listed in Table 2 were initially considered candidate variables. Categorical variables were treated as factors and converted into indicator variables when constructing the design matrix, whereas continuous and ordinal variables were standardized using z-score transformation before model fitting. Because standardization was performed during data preprocessing, additional standardization within glmnet was disabled.

LASSO logistic regression was fitted using an L1 penalty (*α* = 1). The penalty parameter (*λ*) was determined using 10-fold cross-validation based on binomial deviance, with 200 candidate *λ* values evaluated. Both *λ*.min and *λ*.1se were examined, and *λ*.min, corresponding to the minimum cross-validated deviance, was used as the criterion for final variable selection. Variables with non-zero coefficients at *λ*.min were retained and subsequently entered into the multivariable logistic regression model. For categorical variables represented by multiple indicator terms, the variable was considered retained if at least one corresponding coefficient was non-zero; for concise presentation in Table 2, the coefficient with the largest absolute value was reported as the representative LASSO coefficient.

Subsequently, multivariable logistic regression analysis was conducted using SPSS 26.0 to further examine adjusted associations between the selected variables and trajectory class membership. Adjusted odds ratios and 95% confidence intervals were estimated for the associations between the included variables and trajectory class membership.

## 3 Results

### 3.1 General characteristics of ICU survivors

A total of 305 participants completed the baseline assessment at T1. At T2, 301 participants completed the assessment, with 4 participants newly lost to follow-up. At T3, 294 participants completed the assessment, with an additional 7 participants lost to follow-up, resulting in 11 cumulative losses. At T4, 284 participants completed the assessment, with an additional 10 participants lost to follow-up, resulting in 21 cumulative losses. The main reasons for loss to follow-up were refusal to continue participation or inability to establish effective contact after hospital discharge. Ultimately, 284 participants completed all four assessments and were included in the primary complete-case analysis, corresponding to a completion rate of 93.11%.

To assess potential attrition bias, baseline age, sex, primary diagnosis, and T1 PDI total score were compared between participants who completed all four assessments (*n* = 284) and those who did not (*n* = 21). No statistically significant differences were observed between the two groups for these measured baseline characteristics (all *P* > 0.05), providing no clear evidence of differential attrition with respect to these variables.

All participants were enrolled according to the same predefined inclusion and exclusion criteria, and no additional exclusion criteria based on demographic or clinical characteristics were applied during follow-up or final analysis. Ultimately, 284 patients completed assessments at all four time points, resulting in an effective follow-up rate of 93.11%.

In this study, the calculated theoretical sample size was 227 participants. To enhance the robustness of the study results, all 284 patients who completed the longitudinal assessments were included in the final analysis. Among the 284 patients, 147 were male and 137 were female, with a mean age of 45.42 ± 16.25 years. The mean PDI total scores at T1, T2, T3, and T4 were 50.21 ± 23.84, 45.57 ± 20.78, 35.04 ± 14.56, and 27.29 ± 5.61, respectively.

### 3.2 Latent class growth analysis of PDI-measured dignity-related distress trajectories

To explore heterogeneity in longitudinal PDI scores after ICU transfer, this study employed latent class growth analysis (LCGA) based on PDI scores measured at four time points (T1: at ICU transfer, T2: 3 days after transfer, T3: 7 days after transfer, and T4: 14 days after transfer). Models with one to five latent classes were sequentially estimated. The model fit indices and class characteristics of different solutions are presented in Table 1.

**Table 1:** Model fit indices for latent class growth analysis with different class solutions.

Model	AIC	BIC	aBIC	Entropy	*P* LMR BLRT	Class Probability
1	9,262.542	9,291.733	9,266.365			
2	8,126.217	8,166.356	8,131.474	0.994	0.004 ＜0.001	0.739/0.261
3	7,611.730	7,677.411	7,620.332	0.998	0.015 ＜0.001	0.737/0.028/0.235
4	7,304.900	7,388.827	7,315.893	0.991	0.055 ＜0.001	0.721/0.172/0.028/0.079
5	7,212.069	7,314.240	7,225.451	0.958	0.296 ＜0.001	0.627/0.157/0.078/0.029/0.109

The AIC, BIC, and aBIC values progressively decreased as the number of latent classes increased, indicating improved statistical fit with more complex class solutions. However, model selection was not based solely on information criteria. The two-class model showed high classification accuracy (entropy = 0.994), significant LMR and BLRT results, and two adequately sized classes accounting for 73.9% and 26.1% of the sample, respectively.

Although the three-class model showed slightly higher entropy (0.998) and a significant LMR test, it introduced a very small class comprising only 2.8% of the sample, while the other two classes remained broadly similar in proportion to the two major classes identified in the two-class solution. This suggested that the additional class primarily represented further subdivision of the sample rather than a sufficiently stable subgroup for subsequent class-membership analyses.

Further increasing the number of classes did not provide more convincing support for additional complexity. The four-class solution retained a very small class of 2.8%, and the LMR test was no longer statistically significant (*P* = 0.055). Similarly, the five-class solution contained another small class of 2.9%, showed a further decrease in entropy to 0.958, and had a non-significant LMR test (*P* = 0.296). Thus, although the information criteria continued to decrease, the four- and five-class solutions resulted in increasingly fragmented class structures without consistent support from the likelihood-ratio tests.

Taking into account the information criteria, likelihood-ratio tests, entropy, class proportions, classification certainty, parsimony, stability, and clinical interpretability, the two-class solution was therefore retained as the final model. The final two classes included 210 participants (73.94%) and 74 participants (26.06%), with average posterior class probabilities of 0.998 and 0.999, respectively. Entropy was interpreted as an indicator of classification precision rather than as a stand-alone criterion for determining the optimal number of classes. Detailed model fit indices and trajectory patterns across different latent class models are presented in Table 1 and Figure 2.

**Figure 2:**
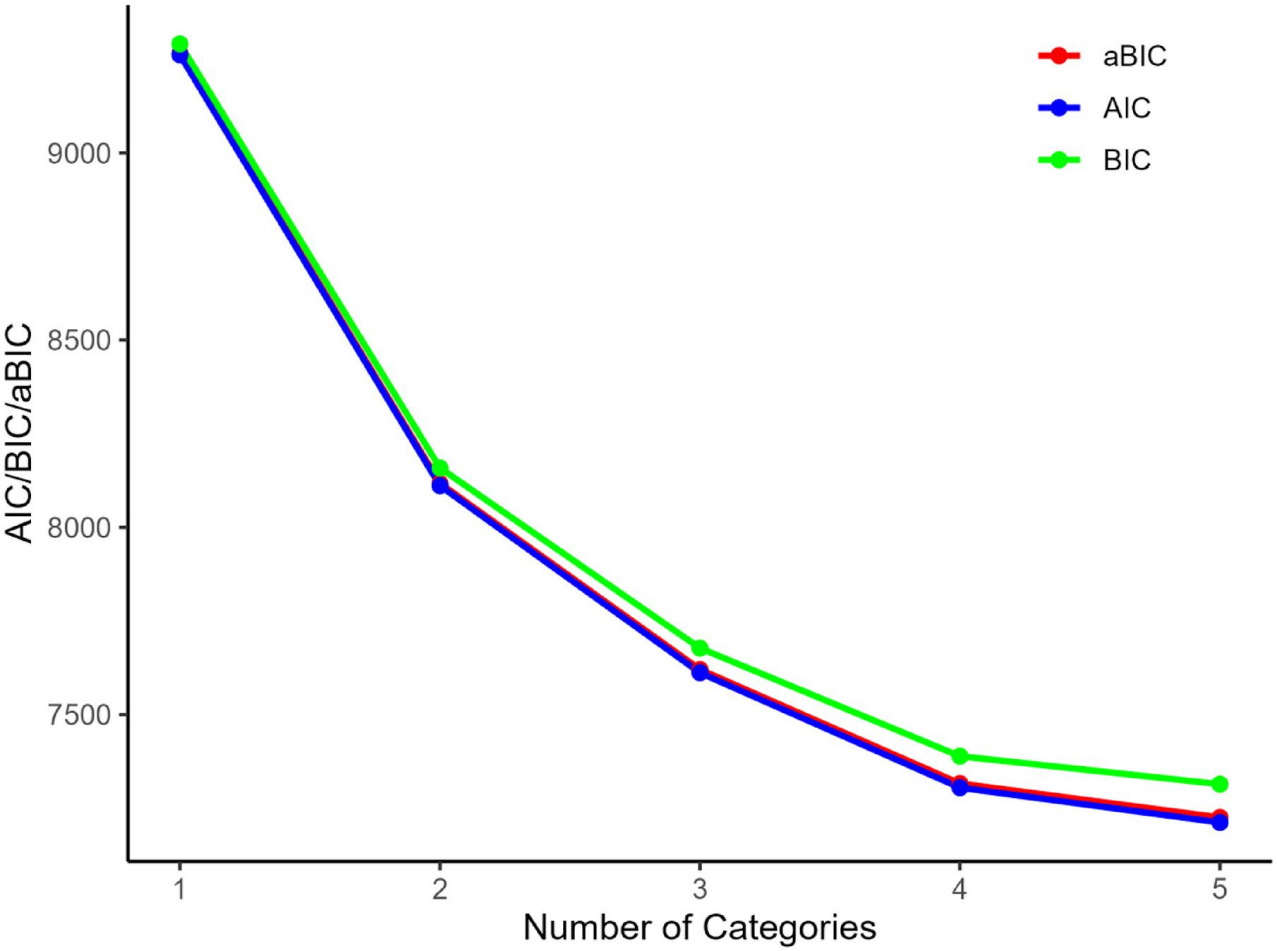
Elbow plot for model selection of PDI-measured dignity-related distress trajectories in ICU survivors.

### 3.3 Identification and characterization of PDI-measured dignity-related distress trajectories

Based on the results of the latent class growth analysis, two distinct trajectories of PDI-measured dignity-related distress were identified (see Figure 3). The trajectories demonstrated different patterns of change across the four follow-up time points (T1: at ICU transfer, T2: 3 days after transfer, T3: 7 days after transfer, and T4: 14 days after transfer), indicating heterogeneity in the recovery process among ICU survivors.

**Figure 3:**
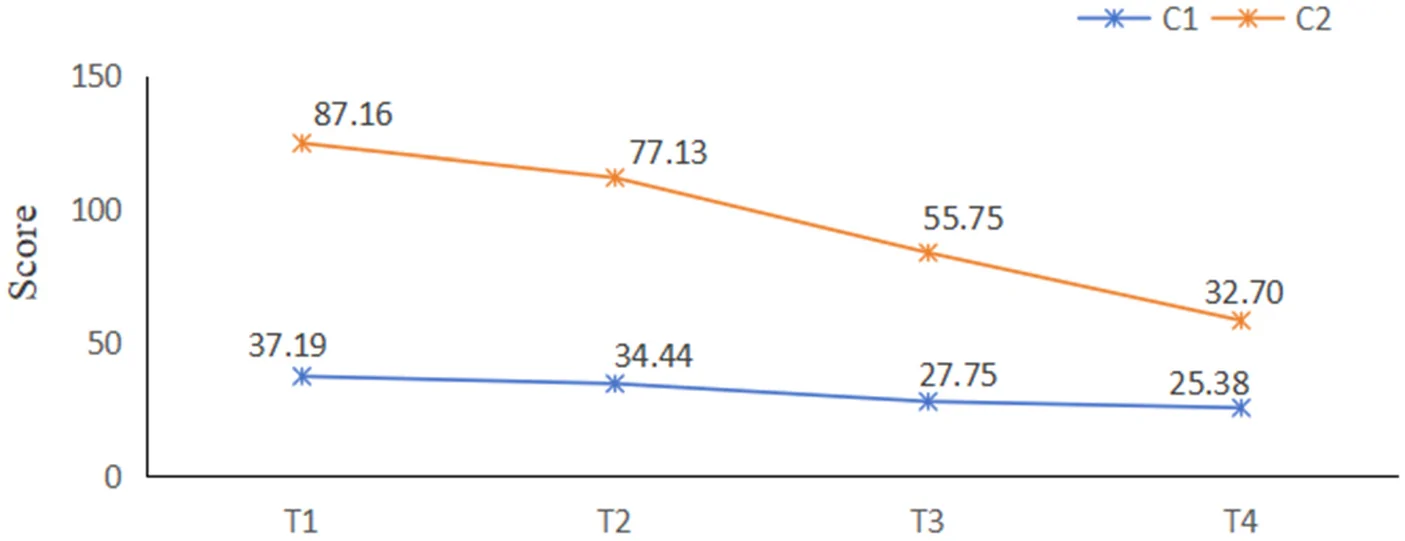
Observed mean PDI scores across four assessment points by latent trajectory class.

Class 1 (C1): Low-level gradual improvement trajectory. This class included 210 participants (73.94%). Patients in this class exhibited relatively low PDI scores throughout the follow-up period, with observed mean scores decreasing from 37.19 at T1 to 34.44 at T2, 27.75 at T3, and 25.38 at T4. In the quadratic LCGA model, the estimated intercept was 37.790, the linear slope was −1.789 (*P* < 0.001), and the quadratic term was 0.064 (*P* < 0.001). These estimates indicate an overall decrease in PDI-measured dignity-related distress, with the rate of decline gradually slowing over time.

Class 2 (C2): High-level rapid improvement trajectory. This class included 74 participants (26.06%). Patients in this class showed markedly higher PDI scores at ICU transfer, with observed mean scores decreasing from 87.16 at T1 to 77.13 at T2, 55.75 at T3, and 32.70 at T4. The estimated intercept was 88.372, the linear slope was −4.948 (*P* < 0.001), and the quadratic term was 0.069 (*P* = 0.001). This pattern indicates a pronounced early decrease in PDI-measured dignity-related distress followed by a gradual deceleration in the rate of improvement. The average posterior probabilities for the two classes were 0.998 and 0.999, respectively, indicating high classification certainty.

Overall, although PDI-measured dignity-related distress decreased over time in both trajectories, patients in Class 2 experienced substantially higher initial symptom levels and a markedly faster rate of improvement compared with those in Class 1.

### 3.4 LASSO selection of candidate variables associated with trajectory class membership

A total of 30 candidate variables were included in the LASSO regression analysis to identify variables associated with trajectory class membership. Ten-fold cross-validation yielded *λ*.mi*n* = 0.007144128 and *λ*.1se = 0.07928213. At *λ*.min, 18 original candidate variables retained non-zero coefficients, whereas five variables were retained at *λ*.1se. Final variable selection was based on *λ*.min.

The 18 variables retained at *λ*.min were gender, ethnicity, employment status, healthcare insurance type, primary diagnosis, frequency of ICU admissions, enteral nutrition therapy, mechanical ventilation therapy, age, BMI, ICU length of stay, CFS score, education level, number of children, visiting frequency, self-rated family harmony, number of combined systemic dysfunctions, and self-rated sleep quality. All 18 variables were subsequently entered into the multivariable logistic regression model. The LASSO coefficients and variable-retention status are presented in Table 2, while the coefficient profiles and 10-fold cross-validation results are shown in Figures 4, 5.

**Table 2:** Candidate variables and LASSO selection for trajectory class membership.

Variable	Group	Class 1 （*n* = 210, 73.94%)	Class 2 (*n* = 74, 26.06%)	LASSO Coefficient	Selected by LASSO regression
Gender No (%)	Male	104 (49.52)	43 (58.11)	0.011	Yes
	Female	106 (50.48)	31 (41.89)		
Age (years, m ± s)		40.73 ± 13.87	58.73 ± 15.15	0.694	Yes
BMI index		24.13 ± 2.50	23.78 ± 3.72	−0.063	Yes
Ethnicity No (%)	Ethnic han	176 (83.81)	53 (71.62)	0.442	Yes
	Other ethnic groups	34 (16.19)	21 (28.38)		
Marital status No (%)	Married	142 (67.62)	47 (63.51)	0.000	No
	Non-married	68 (32.38)	27 (36.49)		
Religious belief No (%)	None	157 (74.76)	59 (79.73)	0.000	No
	Yes	53 (25.24)	15 (20.27)		
Education level No (%)	Primary and below	18 (8.57)	37 (50.00)	−0.127	Yes
	Junior high school	27 (12.86)	12 (16.22)		
	High school	52 (24.76)	14 (18.92)		
	College and above	113 (53.81)	11 (14.86)		
Primary occupation No (%)	Technical personnel	79 (37.62)	12 (16.22)	0.000	No
	Financial business	44 (20.95)	9 (12.16)		
	Service industry	35 (16.67)	8 (10.81)		
	Agriculture	22 (10.48)	33 (44.59)		
	Others	30 (14.29)	12 (16.22)		
Employment status No (%)	Employed	152 (72.38)	40 (54.05)	−0.305	Yes
	Retired	15 (7.14)	7 (9.46)		
	Unemployed	43 (20.48)	27 (36.49)		
Residence No (%)	Rural	71 (33.81)	48 (64.86)	0.000	No
	Urban	138 (66.19)	26 (35.14)		
Healthcare insurance type No (%)	Urban medical insurance	117 (55.71)	15 (20.27)	−0.455	Yes
	Employee medical insurance	76 (36.19)	41 (55.41)		
	Out-of-pocket payment	10 (4.76)	6 (8.11)		
	Others	7 (3.33)	12 (16.22)		
Number of children No (%)	0	56 (26.67)	5 (6.76)	0.308	Yes
	1	102 (48.57)	22 (29.74)		
	2	42 (20.00)	20 (27.03)		
	＞2	10 (4.76)	27 (36.49)		
Self-rated family harmony No (%)	Very harmonious	121 (57.62)	17 (22.97)	−0.154	Yes
	Moderately harmonious	73 (34.76)	39 (52.70)		
	Not harmonious	16 (7.62)	18 (24.32)		
Self-perceived financial pressure No (%)	High	11 (5.24)	11 (14.86)	0.000	No
	Moderate	99 (47.14)	48 (64.86)		
	None	100 (47.62)	15 (20.27)		
APACHE II score （score,m ± s）		33.36 ± 8.85	31.63 ± 9.30	0.000	No
CFS score (score, m ± s)		2.52 ± 1.27	3.79 ± 1.57	−0.228	Yes
Type of ICU No (%)	Medical ICU	115 (54.76)	21 (28.38)	0.000	No
	Surgical ICU	74 (35.24)	26 (35.14)		
	Emergency ICU	12 (5.71)	20 (27.03)		
	Other ICUs	9 (4.29)	7 (9.46)		
ICU length of stay （days, m ± s）		3.60 ± 2.81	10.09 ± 5.71	0.634	Yes
Frequency of ICU admissions No (%)	Once	157 (74.76)	37 (50.00)	0.094	Yes
	Twice	26 (12.38)	14 (18.92)		
	More than twice	27 (12.86)	23 (31.08)		
Primary visitor No (%)	Spouse	107 (50.95)	26 (35.14)	0.000	No
	Parents	72 (34.29)	10 (13.51)		
	Children	26 (12.38)	36 (48.65)		
	Others	5 (2.38)	2 (2.70)		
Visiting frequency No (%)	At least once a day	166 (79.05)	24 (32.43)	0.036	Yes
	About once every 2 days	27 (12.86)	16 (21.62)		
	About once every 3 days	6 (2.86)	18 (24.32)		
	More than once every 3 days	11 (5.24)	16 (21.62)		
Primary diagnosis No (%)	Nervous system	40 (19.05)	26 (35.14)	0.709	Yes
	Respiratory system	18 (8.57)	23 (31.08)		
	Circulatory system	79 (37.62)	12 (16.22)		
	Digestive system	40 (19.05)	2 (2.70)		
	Other systemic	33 (15.71)	11 (14.86)		
Number of combined systemic dysfunctions No (%)	1 system	127 (60.48)	19 (25.68)	0.506	Yes
	2 systems	80 (38.09)	26 (35.14)		
	3 systems	3 (1.43)	22 (29.73)		
	＞3 systems	0 (0.00)	7 (9.46)		
Enteral nutrition therapy No (%)	No	26 (12.38)	20 (27.03)	0.909	Yes
	Yes	184 (87.62)	54 (72.97)		
Mechanical ventilation therapy No (%)	No	94 (44.76)	17 (22.97)	−0.156	Yes
	Yes	116 (55.24)	57 (77.03)		
Sedation and (or) analgesia therapy No (%)	No	51 (24.29)	7 (9.46)	0.000	No
	Yes	159 (75.71)	67 (90.54)		
Protective restraint No (%)	No	99 (47.14)	17 (22.97)	0.000	No
	Yes	111 (52.86)	57 (77.03)		
Indwelling catheter No (%)	No	17 (8.10)	1 (1.35)	0.000	No
	Yes	193 (91.90)	73 (98.65)		
Gastric tube placement No (%)	No	73 (34.76)	6 (8.11)	0.000	No
	Yes	137 (65.24)	68 (91.89)		
Self-rated sleep quality No (%)	Very poor	87 (41.43)	32 (43.24)	−0.231	Yes
	Poor	70 (33.33)	34 (45.95)		
	Fair	43 (20.48)	8 (10.81)		
	Good	10 (4.76)	0 (0.00)		

Class 1 represents the low-level gradual improvement trajectory, and Class 2 represents the high-level rapid improvement trajectory. Class-specific values are presented descriptively. Variables marked as “Yes” retained non-zero coefficients at *λ*.min and were subsequently entered into the multivariable logistic regression model. For categorical variables represented by multiple indicator terms, the coefficient with the largest absolute value is presented as the representative LASSO coefficient. For multi-level categorical variables, this representative coefficient is provided only to summarize variable retention in the LASSO procedure and should not be interpreted as a complete effect estimate for the variable.

**Figure 4:**
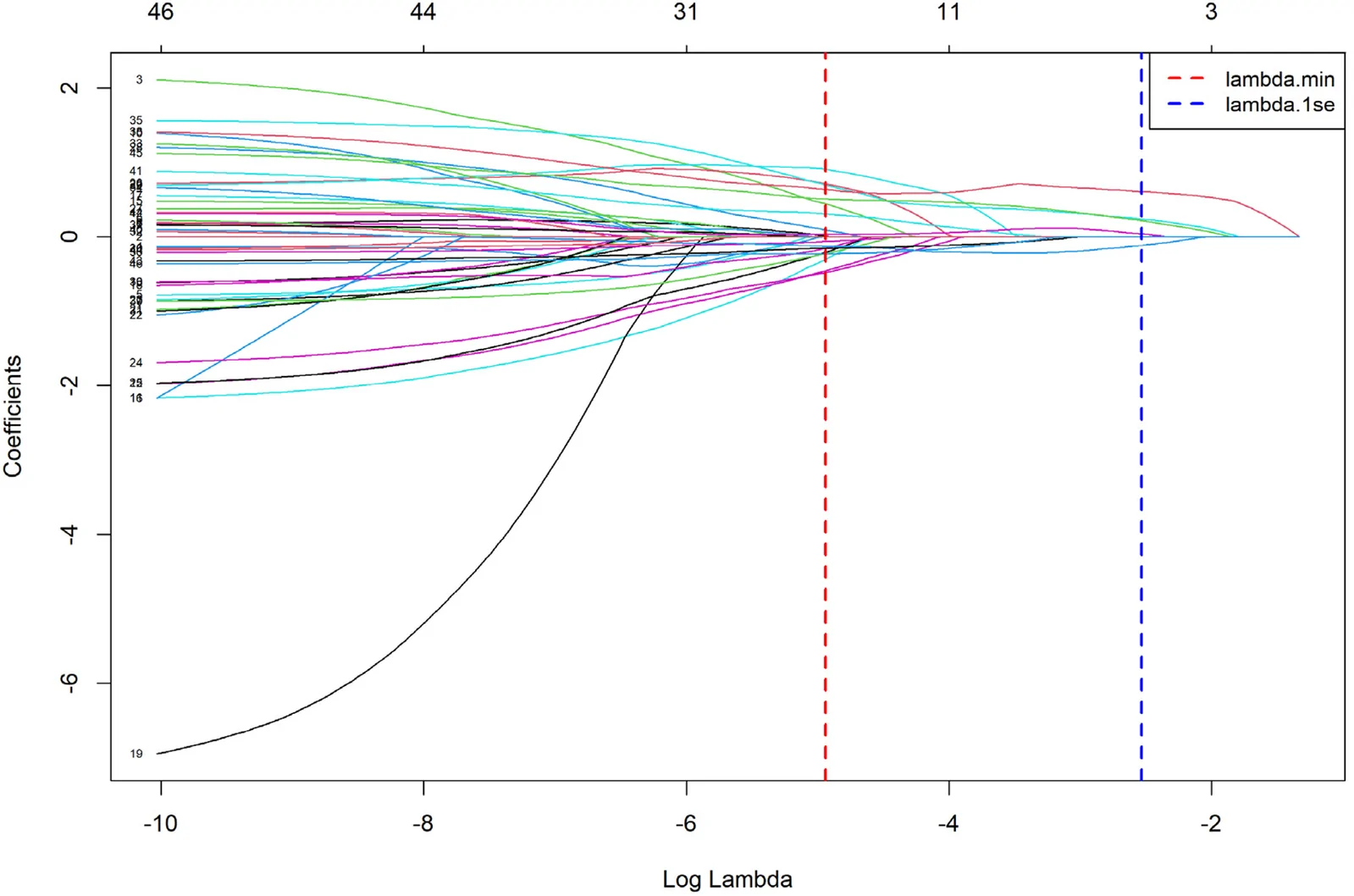
LASSO coefficient profiles of variables associated With trajectory class membership.

**Figure 5:**
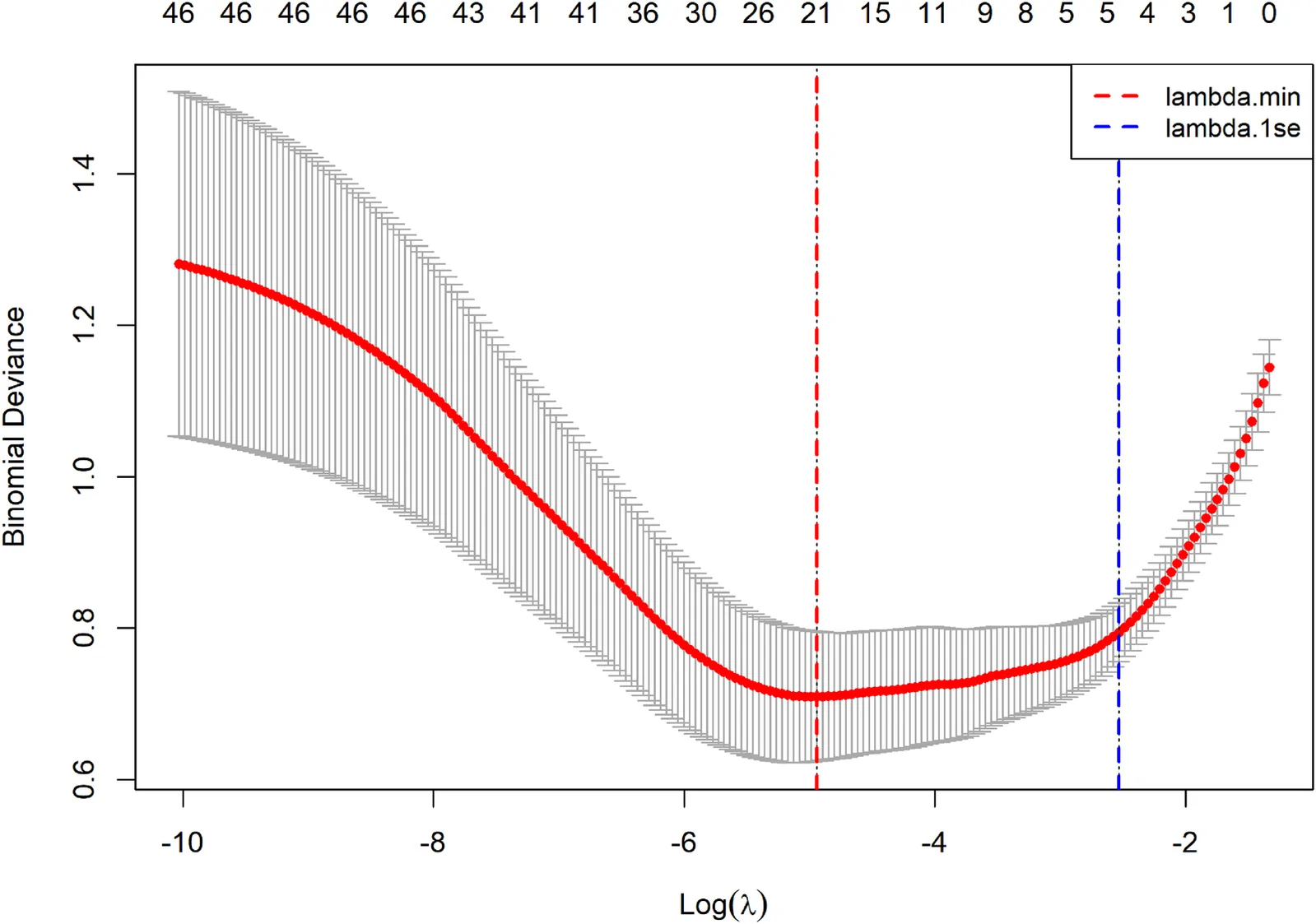
Cross-validation plot for selecting the optimal penalty parameter (*λ*) in the LASSO model.

### 3.5 Multivariable logistic regression analysis of factors associated with trajectory class membership

To further examine factors associated with latent trajectory class membership after multivariable adjustment, multivariable logistic regression analysis was performed with Class 2 versus Class 1 trajectory membership as the dependent variable and Class 1 as the reference category. All 18 variables retained by LASSO regression with non-zero coefficients at *λ*.min were simultaneously entered into the multivariable logistic regression model. Accordingly, the odds ratios derived from this model represent adjusted associations after simultaneous control for the other variables included in the model. These variables were gender, ethnicity, employment status, healthcare insurance type, primary diagnosis, frequency of ICU admissions, enteral nutrition therapy, mechanical ventilation therapy, age, BMI, ICU length of stay, CFS score, education level, number of children, visiting frequency, self-rated family harmony, number of combined systemic dysfunctions, and self-rated sleep quality. All 18 variables were retained in the multivariable model regardless of their statistical significance, and statistical significance was used only for interpretation of the adjusted associations rather than for further variable elimination. For concise presentation and ease of interpretation, Table 3 presents only variables showing statistically significant adjusted associations with trajectory class membership. Prior to the multivariable analysis, multicollinearity diagnostics were performed for all independent variables included in the model. The results showed that the variance inflation factor (VIF) values for all variables were less than 5 and the tolerance values were greater than 0.1, indicating that no significant multicollinearity existed among the independent variables (13). Therefore, multivariable logistic regression analysis was considered appropriate.

**Table 3:** Multivariable logistic regression analysis of factors associated with membership in class 2 versus class 1 PDI-measured dignity-related distress trajectory among ICU survivors.

Variable	Category	B	SE B	P	OR	95%CI
Age		0.098	0.031	0.002	1.103	1.038–1.173
CFS score		−0.580	0.260	0.026	0.560	0.336–0.932
Number of combined systemic dysfunctions		0.779	0.339	0.021	2.180	1.123–4.232
Primary diagnosis	Nervous system	—	—	—	—	—
	Respiratory system	−1.057	0.716	0.140	0.348	0.085–1.414
	Circulatory system	−1.729	0.912	0.058	0.177	0.030–1.060
	Digestive system	−2.261	0.750	0.003	0.104	0.024–0.453
	Other systemic	−2.150	0.942	0.022	0.116	0.018–0.738
ICU length of stay		0.181	0.079	0.021	1.199	1.028–1.398

The overall association of primary diagnosis with trajectory class membership was assessed using an omnibus Wald test (Wald *χ^2^*= 11.518, df = 4, *P* = 0.021). Category-specific *P* values and odds ratios represent Wald comparisons with nervous system diseases as the reference category. The Hosmer–Lemeshow goodness-of-fit test indicated adequate model fit (*χ^2^* = 10.945, *P* = 0.205). Class 1 was used as the reference outcome category. Odds ratios (ORs) represent the odds of membership in Class 2 versus Class 1. OR > 1 indicates higher odds of Class 2 membership, whereas OR < 1 indicates lower odds of Class 2 membership.

The results of the multivariable logistic regression analysis indicated that age, Clinical Frailty Scale (CFS) score, ICU length of stay, number of combined systemic dysfunctions, and primary diagnosis were significantly associated with PDI-measured dignity-related distress trajectory class membership (all *P* < 0.05). Specifically, older age (OR=1.103, 95% CI: 1.038–1.173), longer ICU length of stay (OR=1.199, 95% CI: 1.028–1.398), and a greater number of combined systemic dysfunctions (OR=2.180, 95% CI: 1.123–4.232) were associated with a higher likelihood of being classified into Class 2.In contrast, higher CFS score (OR=0.560, 95% CI: 0.336–0.932) was associated with a lower probability of belonging to Class 2.Regarding primary diagnosis, with nervous system diseases as the reference category, patients with digestive system diseases (OR=0.104, 95% CI: 0.024–0.453) and other systemic diseases (OR=0.116, 95% CI: 0.018–0.738) were more likely to be classified into Class 1. Detailed results are presented in Table 3.

## 4 Discussion

### 4.1 Two exploratory trajectories of PDI-measured dignity-related distress were identified among ICU survivors

Based on latent class growth analysis (LCGA), this study longitudinally examined the developmental trajectories of PDI-measured dignity-related distress among ICU survivors. The results identified two longitudinal trajectories of PDI-measured dignity-related distress during the recovery process: a low-level gradual improvement trajectory and a high-level rapid improvement trajectory. Patients in the low-level gradual improvement trajectory exhibited relatively lower levels of PDI-measured dignity-related distress at the time of ICU transfer, followed by a gradual decline during the follow-up period, although the rate of improvement was relatively slow. In contrast, patients in the high-level rapid improvement trajectory presented with markedly higher levels of PDI-measured dignity-related distress at ICU transfer but experienced a rapid decline in symptom severity during follow-up, indicating a more pronounced recovery trend. These findings suggest that substantial individual differences exist in the psychological recovery process among ICU survivors, with different patient subgroups demonstrating distinct patterns in both initial severity and the rate of change in PDI-measured dignity-related distress.

Notably, the two trajectory classes differed substantially in PDI-measured dignity-related distress at T1, in addition to showing different patterns of change over time. Therefore, the identified classes should be interpreted as data-driven trajectory patterns rather than definitive longitudinal recovery phenotypes. Although the significant linear and quadratic growth parameters indicate that the classes differed in their subsequent symptom evolution, the possibility that the class solution partly reflects stratification by baseline symptom severity cannot be excluded. This interpretation is particularly important given that only four assessment occasions were available during the first 14 days after ICU transfer. Future studies incorporating more repeated measurements and alternative growth mixture modeling approaches are needed to determine whether these patterns represent reproducible recovery phenotypes.

Although models with additional latent classes demonstrated better statistical fit, the additional classes mainly represented relatively small subgroups, which may limit their clinical interpretability and stability. Therefore, the two-class solution was considered more appropriate because it balanced statistical performance with meaningful clinical differentiation. The two-class solution was therefore considered the most parsimonious and clinically interpretable representation of the observed data. These data-driven trajectory patterns may help characterize differences in early dignity-related recovery and may provide a useful basis for identifying patients with different supportive care needs.

To better interpret these trajectory patterns, it is also important to consider the timing of symptom assessments. The selection of the four assessment time points in this study was designed to capture the dynamic changes in PDI-measured dignity-related distress symptoms during the early post-ICU transition period. The first two weeks after ICU transfer represent a critical phase in which patients experience substantial changes in physical condition, treatment environment, and psychological adaptation after critical illness. Therefore, repeated assessments at ICU transfer, 3 days, 7 days, and 14 days after ICU transfer enabled the observation of early symptom evolution and facilitated the identification of heterogeneous trajectories of PDI-measured dignity-related distress among ICU survivors.

Notably, many patients experience impaired consciousness, severe illness, or sedation during ICU admission, making it difficult to assess their pre-ICU baseline levels of dignity-related distress. Therefore, when interpreting the distinct recovery trajectories, it cannot be ruled out that some patients already had high baseline levels of dignity-related distress prior to ICU admission, and the rapid improvement after ICU transfer may represent a return to their pre-existing baseline. This clarification helps to more objectively understand the early trajectory characteristics of PDI-measured dignity-related distress among ICU survivors.

From the overall trend, PDI-measured dignity-related distress in both trajectory groups gradually decreased over time. This pattern is consistent with the recovery process of critically ill patients, who gradually adapt to changes in their physical condition and the surrounding environment during the post-ICU period (13). After transfer from the ICU, patients are progressively removed from highly invasive treatment environments, their physical functions gradually improve, and communication with family members and healthcare providers increases (14). These factors may contribute to alleviating the psychological stress and dignity-related distress experienced during the ICU stay. However, patients with higher initial symptom levels may have undergone more invasive treatments during the acute phase of illness, such as mechanical ventilation, physical restraint, or sedation and analgesia therapy. These interventions may increase patients’ feelings of dependency and loss of control, thereby contributing to more severe experiences of dignity-related distress (15).

The findings of this study further highlight the clinical importance of identifying different trajectories of PDI-measured dignity-related distress among ICU survivors. For patients classified into the high-level rapid improvement trajectory, early psychological support and dignity-conserving care may represent potential approaches for future evaluation, particularly during the initial period after ICU transfer. For patients in the low-level gradual improvement trajectory, the potential value of continued assessment during recovery also warrants investigation. Overall, these findings suggest that repeated assessment of psychological status and dignity-related experiences after ICU transfer, together with individualized supportive-care approaches, may be useful areas for future clinical research.

### 4.2 Factors associated With PDI-measured dignity-related distress trajectory class membership in ICU survivors

#### 4.2.1 Age

The results of this study indicate that age was associated with PDI-measured dignity-related distress trajectory membership. Multivariate logistic regression analysis showed that older patients were more likely to be classified into Class 2 (OR=1.103, *P* < 0.05). Combined with trajectory analysis results, patients in Class 2 exhibited higher initial PDI-measured dignity-related distress at the time of ICU transfer but experienced a relatively rapid decline in symptom severity during the follow-up period. This finding suggests that older patients may experience greater dignity-related distress during the acute stage of illness, while their psychological status may gradually improve as the recovery process progresses (16).

On the one hand, older patients are more likely to undergo multiple invasive treatments during ICU care, such as mechanical ventilation, sedation and analgesia therapy, or physical restraint. Although these interventions are clinically necessary, they may temporarily deprive patients of autonomy and control, leading to feelings of dependency, helplessness, and questioning of self-worth. In the ICU environment, where privacy is limited and daily activities often require assistance from healthcare providers or family members, this shift in personal roles may further reinforce older patients’ perceptions of declining functional ability, thereby increasing their experience of dignity-related distress at the time of ICU transfer (17).

One possible explanation for this pattern is that older patients may have accumulated greater life experience and coping experience that could facilitate adaptation to health-related changes during recovery (18). Family and social support may also contribute to this pattern, as older patients may receive emotional and caregiving support from spouses, children, or other family members during hospitalization (19). However, psychological adaptability, coping capacity, and family support were not directly measured in the present study. Therefore, these explanations should be regarded as hypotheses for interpreting the observed association rather than established mechanisms.

These findings suggest that the dignity-related experiences of older ICU survivors may warrant further attention in future studies. Repeated assessment of dignity-related distress and supportive-care approaches could be evaluated alongside physical recovery during the early post-ICU period. Interventions such as respectful communication, protection of patient privacy, and encouragement of gradual participation in self-care activities may help patients rebuild confidence in their abilities. Furthermore, enhancing family involvement and emotional support may also facilitate psychological adaptation and accelerate the recovery of dignity-related distress.

#### 4.2.2 CFS score

The results of this study showed that the CFS score was associated with trajectory class membership. Multivariate logistic regression analysis indicated that patients with higher CFS scores were more likely to be classified into the low-level gradual improvement trajectory (Class 1) (OR=0.560, *P* < 0.05).

The CFS score was used to assess pre-morbid frailty status before ICU admission (20). Counterintuitively, a higher score reflected poorer baseline physical function but was associated with milder PDI-measured dignity-related distress. In this study, patients with greater pre-morbid frailty paradoxically exhibited lower initial PDI-measured dignity-related distress at ICU transfer, following a trajectory of low-level gradual improvement.

One possible explanation is that patients with greater pre-morbid frailty may have had more previous experience with functional limitations, physical dependence, and changes in daily roles, which could potentially shape their psychological response to critical illness and subsequent recovery (21). However, adaptation to illness, acceptance of dependence, and psychological resilience were not directly assessed in the present study. Therefore, this interpretation remains hypothesis-generating, and the mechanisms underlying the observed association between frailty and PDI-measured dignity-related distress trajectories require further investigation.

The observed association between pre-morbid frailty and trajectory class membership suggests that frailty status may be relevant when investigating dignity-related distress during early post-ICU recovery. Future studies could examine whether patients with different levels of pre-morbid frailty have distinct supportive-care needs and whether tailored psychological support is beneficial.

#### 4.2.3 Number of combined systemic dysfunctions

The number of combined systemic dysfunctions was associated with trajectory class membership. Multivariate logistic regression analysis showed that patients with a greater number of combined systemic dysfunctions were more likely to be classified into Class 2 (OR=2.180, *P* < 0.05). Consistent with trajectory analysis, patients in Class 2 presented with higher levels of PDI-measured dignity-related distress at the time of ICU transfer, followed by a relatively rapid decline in symptom severity during the follow-up period.

This indicator does not equate to illness severity, but reflects disease involvement, treatment complexity, and intervention intensity (22), which are directly relevant to the study objectives. On the one hand, patients with multiple systemic dysfunctions usually require complex treatment strategies, including life-support measures, continuous monitoring, and multiple indwelling devices. Although essential for maintaining life, these interventions may increase dependence on caregivers, restricting autonomy and potentially increasing early dignity-related distress (23).

On the other hand, as patients’ conditions gradually stabilize and they are transferred out of the ICU environment, improvements in physical function and reductions in medical interventions may contribute to improvements in psychological well-being. After leaving the highly technical and restrictive ICU environment, patients are often able to gradually restore communication with family members and resume certain daily activities. These positive changes may help alleviate the psychological stress experienced during the acute phase of illness and promote the recovery of dignity-related distress (24). Consequently, although patients with multiple systemic dysfunctions may present with higher levels of PDI-measured dignity-related distress at ICU transfer, their symptoms may decrease more rapidly during the follow-up period, forming a trajectory characterized by high initial levels followed by rapid improvement. It should also be noted that invasive procedures such as mechanical ventilation, gastric tube, and urinary catheter were included as treatment-related variables, and the number of combined systemic dysfunctions may further reflect treatment complexity and the likelihood of invasive procedures. Although central venous catheterization, dialysis, and drainage were not separately recorded because of their dynamic and multi-outcome nature during ICU stay, these variables collectively provide direct and indirect information on the burden of invasive procedures. They were included in the multivariable model, thus partially accounting for treatment-related factors that may be associated with trajectory membership.

Therefore, in clinical practice, particular attention should be given to ICU survivors with multiple systemic dysfunctions. Healthcare providers should not only focus on physical recovery but also pay close attention to the psychological burden and dignity-related experiences associated with complex medical treatments. Strengthening communication between healthcare providers and patients, minimizing unnecessary restrictive measures, and encouraging gradual participation in self-care activities may help alleviate psychological stress and facilitate the restoration of patients’ sense of dignity.

#### 4.2.4 Primary diagnosis

Category-specific differences in trajectory class membership were observed across primary diagnosis groups. In ICU settings, common primary diagnoses typically include neurological diseases, respiratory diseases, circulatory diseases, digestive system diseases, and other systemic conditions. These different disease categories vary considerably in terms of clinical manifestations, treatment approaches, and their impact on patients’ functional status, which may consequently be associated with differences in patients’ psychological experiences and perceptions of dignity.

For patients with neurological diseases, these conditions are often associated with a relatively high risk of disability and may be accompanied by impaired consciousness, motor dysfunction, speech difficulties, and swallowing disorders (25). Such functional impairments can significantly affect patients’ ability to perform daily activities and may lead to a greater sense of dependency and loss of control during the course of treatment. When patients are unable to independently perform basic daily activities or express their needs effectively, their sense of self-worth and social role identity may be substantially challenged, thereby increasing the likelihood of experiencing dignity-related distress. In addition, patients with neurological diseases often require more intensive monitoring and nursing care during ICU treatment, such as continuous neurological assessment, prolonged bed rest, and multiple invasive procedures. These factors may further increase the psychological burden experienced by patients during the early stage of illness (26).

In contrast, patients with digestive system diseases generally maintain intact consciousness and cognitive function during the acute phase. As a result, these patients are often able to communicate effectively with healthcare providers and family members and have a clearer understanding of their illness and treatment process. This preserved ability for communication and participation in decision-making may help maintain a certain degree of autonomy and control, thereby mitigating the psychological distress associated with dignity-related distress.

The category of other systemic diseases generally includes conditions that are not classified within the major organ systems mentioned above, such as postoperative orthopedic conditions (e.g., fracture surgery), certain urological disorders, or other surgical conditions requiring short-term intensive monitoring. Although these patients may experience postoperative pain, temporary mobility limitations, or short-term intensive care monitoring, their illnesses usually do not directly impair consciousness or core neurological functions (27). Consequently, patients in this group often retain relatively intact cognitive abilities, social interaction, and emotional expression, which may help them maintain a sense of autonomy and personal value during treatment. It should also be noted that patients within this category may differ in admission route, surgical urgency, and the extent of preoperative communication and education. In clinical practice, patients undergoing elective surgery are often well counseled by surgeons and anesthesiologists before the procedure, which may provide them with greater psychological preparation and help them maintain a sense of control, whereas patients emergently admitted to the ICU may have limited opportunity for such preparation and may therefore be more vulnerable to dignity-related distress.

Therefore, when neurological diseases were used as the reference category in the present study, patients with digestive system diseases and other systemic diseases were more likely to belong to the low-level gradual improvement trajectory(Class 1). These findings suggest that particular attention should be paid to patients with neurological diseases when developing psychological care and dignity-conserving interventions for ICU survivors. Healthcare providers should strengthen the assessment of patients’ psychological status and dignity-related experiences, and provide supportive interventions such as enhancing communication, encouraging participation in rehabilitation activities, and offering emotional support. Such approaches may help patients gradually restore their sense of self-worth and reduce dignity-related distress.

#### 4.2.5 ICU length of stay

ICU length of stay was associated with trajectory class membership. Multivariate logistic regression analysis showed that a longer ICU stay was associated with a higher likelihood of being classified into Class 2(OR=1.199, *P* < 0.05).

A longer ICU stay may indicate a more complex disease course and greater exposure to intensive treatment. Patients with prolonged ICU admission experience more invasive procedures, prolonged bed rest, and higher dependence on life support and nursing care. These factors reduce autonomy and exacerbate feelings of helplessness, leading to more severe PDI-measured dignity-related distress at ICU transfer. A longer ICU stay is also accompanied by stricter environmental restrictions and reduced family interaction (28). Limited visiting hours, continuous monitoring, and frequent medical interventions further undermine patients’ sense of control and self-worth (29). These experiences result in higher initial PDI-measured dignity-related distress, which then alleviates rapidly after ICU transfer.

The observed association with ICU length of stay suggests that patients with prolonged ICU exposure may represent a group of interest for future studies of dignity-conserving supportive care. Potential approaches, such as preserving autonomy, facilitating family communication, and minimizing unnecessary restrictive measures, warrant further evaluation in prospective intervention studies.

### 4.3 Clinical implications

From a translational perspective, these findings suggest a feasible stepped approach during the ICU-to-ward transition. PDI-measured dignity-related distress could potentially be assessed by trained ward nurses at or shortly after ICU transfer, with repeated assessment during early ward recovery for patients showing greater initial distress or persistent symptoms. Basic dignity-conserving measures, including respectful communication, protection of privacy, preservation of autonomy, facilitation of family communication, and encouragement of participation in self-care and rehabilitation activities, could be incorporated into routine nursing care. Patients with persistently high or worsening PDI scores, severe distress on individual PDI items, or continuing difficulty adapting despite routine supportive care could be considered for further multidisciplinary assessment, including psychological, psychiatric, rehabilitation, or other supportive services as clinically appropriate. Importantly, the present study was not designed to establish a validated PDI cutoff for initiating intervention in ICU survivors. Therefore, we do not propose a fixed numerical threshold; rather, persistent or worsening dignity-related distress on repeated assessment may warrant further clinical evaluation. Future studies are needed to establish clinically validated screening thresholds and to evaluate the effectiveness of specific dignity-conserving interventions during the ICU-to-ward transition.

## 5 Conclusion

In conclusion, this exploratory prospective cohort study identified two distinct trajectories of PDI-measured dignity-related distress among communicative and cognitively intact ICU survivors during the first 14 days after ICU transfer. Most participants showed a gradual reduction in PDI scores, whereas a smaller subgroup had higher initial scores followed by more rapid improvement. These findings suggest heterogeneity in dignity-related psychological recovery after critical illness and may help generate hypotheses for future research. Given the single-centre design, short follow-up period, eligibility restrictions, and measurement limitations, the identified trajectory classes should not be interpreted as validated clinical risk categories and should not be generalized to all ICU survivors.

## 6 Limitations

This study has several limitations. First, the single-centre design and eligibility criteria may limit generalizability, particularly because patients with cognitive, neuropsychiatric, or communication impairments were underrepresented. Second, although the PDI demonstrated good internal consistency in the present sample, the study-specific Chinese-language adaptation was not formally validated for construct validity in post-ICU survivors. In addition, longitudinal measurement invariance across the four assessments and equivalence across different administration modes, including face-to-face and telephone-based follow-up, were not examined; therefore, potential mode-related measurement variation cannot be excluded. Third, dignity-related distress was first assessed at ICU transfer without a pre-ICU baseline, and follow-up was limited to four assessments over 14 days. Residual confounding from unmeasured clinical, psychological, or social factors may also remain. Finally, the LCGA findings should be considered exploratory. The identified classes may partly reflect differences in initial PDI scores, and the trajectory solution requires confirmation in larger, multicentre cohorts with longer follow-up and external validation.

## Data Availability

The raw data supporting the conclusions of this article will be made available by the authors, without undue reservation.
